# Deciphering the potential pharmaceutical mechanism of Guzhi Zengsheng Zhitongwan on rat bone and kidney based on the “kidney governing bone” theory

**DOI:** 10.1186/s13018-020-01677-8

**Published:** 2020-04-15

**Authors:** Baojin Yao, Jia Liu, Mei Zhang, Xiangyang Leng, Daqing Zhao

**Affiliations:** 1 Jilin Ginseng Academy, Changchun University of Chinese Medicine, Changchun, 130117 Jilin China; 2 College of Pharmacy, Changchun University of Chinese Medicine, Changchun, 130117 Jilin China; 3 Innovation Practice Center, Changchun University of Chinese Medicine, Changchun, 130117 Jilin China; 4 The Affiliated Hospital of Changchun University of Chinese Medicine, Changchun, 130117 Jilin China

**Keywords:** Chinese medicinal formulation, Guzhi Zengsheng Zhitongwan, Pharmaceutical mechanism, Osteoarthritis, Kidney governing bone

## Abstract

**Background:**

Guzhi Zengsheng Zhitongwan (GZZSZTW) is an effective Chinese medicinal formulation for the treatment of osteoarthritis (OA) designed according to the “kidney governing bone” theory, which has been widely used as a golden guide for treating bone and cartilage diseases in traditional Chinese medicine. The aim of this study was to explore the molecular mechanism underlying its effects on the bone and kidney.

**Methods:**

Preparation and quality control were performed as previously described. Since GZZSZTW is orally administered in the form of pills prepared in boiled water, the Chinese materia medica (CMM) mixture of this formula was extracted with distilled water by a reflux method and was then filtered through a 0.45-μm Hollow Fiber Cartridge (GE Healthcare, USA). The filtrate was freeze-dried by a Heto PowerDry LL3000 Freeze Dryer (Thermo, USA) and stored at − 80 °C. The effects of GZZSZTW on gene expression and regulation of both kidney and bone tissues were investigated using a state-of-the-art RNA-seq technology.

**Results:**

We demonstrated that GZZSZTW could enhance kidney function and suppress bone formation and resorption by modulating the activities of osteoblast and osteoclast, and might subsequently contribute to the inhibition of osteophyte formation during the process of OA. These effects might be achieved by the synergistic interactions of various herbs and their active components in GZZSZTW, which increased the expression levels of functional genes participating in kidney function, regulation, and repair, and then decreased the expression levels of genes involved in bone formation and resorption. Thus, our findings were consistent with the “kidney governing bone” theory, which has been widely used as a guide in clinical practice for thousands of years.

**Conclusions:**

This study has deepened the current knowledge about the molecular effects of GZZSZTW on bone and kidney regulation. Furthermore, this study might be able to provide possible strategies to further prevent and treat joint diseases by using traditional Chinese medicinal formulations following the “kidney governing bone” theory.

## Background

The theory of “kidney governing bone” has been widely used as a golden guide in traditional Chinese medicine (TCM) for treating bone and cartilage diseases in clinic for many centuries. According to the definition of kidney in TCM concept, which is distinct from the western medicine, kidney is a comprehensive organ that is not only considered as an anatomical structure. In TCM, the kidney is associated with the gate of vitality (ming men), which show multiple interconnected functions including storing and controlling “essence” and “qi”, and determining growth, development, reproduction, and aging. Therefore, kidney is in charge of the skeleton’s growth, development, and repair [1].

Guzhi Zengsheng Zhitongwan (GZZSZTW), a Chinese medicinal formulation designed by the national medical master professor Bailing Liu, has been used effectively in the Affiliated Hospital of Changchun University of Chinese Medicine for several decades to treat joint diseases such as osteoarthritis (OA). According to Professor Liu’s clinical thought and experience, to cure the kidney is to cure the bone. Therefore, the major components in GZZSZTW are Chinese materia medica (CMM) with functions to tonify the kidney, such as Shu Di (*Rehmannia glutinosa* (Gaertn.) DC*.*), Ying Yang Huo (*Epimedium brevicornu Maxim* (K.S.Hao)), Gu Sui Bu (*Drynaria fortunei* (Kunze ex Mett.) J.Sm*.* (baked)), Suo Yang (*Cynomorium coccineum subsp. songaricum* (*Rupr.*) (J.Léonard)), and Gou Ji (*Cibotium barometz* (*L.*) (J.Sm)).

OA is the most common degenerative joint disease with multiple pathological changes including progressive loss and destruction of articular cartilage, thickening of the subchondral bone, formation of osteophytes, variable degrees of inflammation of the synovium, degeneration of ligaments and menisci of the knee, and hypertrophy of the joint capsule [2]. In addition, pathological changes of adipose tissues such as infrapatellar fat pad (IFP) in the knee joint are considered to be important players in the inflammatory reactions in OA [3]. GZZSZTW has been shown to stimulate chondrocyte proliferation, prevent chondrocyte differentiation, and modulate chondrocyte structure, dynamics, and metabolism by controlling multiple functional genes and proteins [4, 5]. Although it is effective and widely used in the treatment of joint diseases in clinical practice, the precise molecular mechanism underlying the relationship between kidney and bone regulation is still not clearly understood.

In the present study, we performed RNA-seq to analyze the gene expression patterns of kidney and bone in response to the treatment of GZZSZTW in Sprague-Dawley (SD) rats. We demonstrated that GZZSZTW could enhance kidney function and suppress bone formation and resorption by modulating the activities of osteoblast and osteoclast, and might subsequently contribute to the inhibition of osteophyte formation during the process of OA. These effects might be achieved by the synergistic interactions of various herbs and their active components in GZZSZTW, which increased the expression levels of functional genes participating in kidney function, regulation, and repair, and then decreased the expression levels of genes involved in bone formation and resorption.

## Methods

### Preparation of the GZZSZTW aqueous extract

GZZSZTW was obtained from the Affiliated Hospital of Changchun University of Chinese Medicine (Changchun, China). The formulation of GZZSZTW consisted of 7 types of CMM; namely, *Rehmannia glutinosa* (Gaertn.) DC*.*, *Spatholobus suberectus* Dunn, *Epimedium brevicornu Maxim* (K.S.Hao), *Raphanus sativus L.* (Hook. f. & T. Anderson) (baked), *Drynaria fortunei* (Kunze ex Mett.) J.Sm*.* (baked), *Cynomorium coccineum subsp. songaricum* (*Rupr.*) (J.Léonard), and *Cibotium barometz* (*L.*) (J.Sm). Since GZZSZTW is orally administered in the form of pills prepared in boiled water, preparation of GZZSZTW aqueous extract was performed as follows: the CMM mixture of this formula was extracted with distilled water by a reflux method and was then filtered through a 0.45-μm Hollow Fiber Cartridge (GE Healthcare, USA). The filtrate was freeze-dried by a Heto PowerDry LL3000 Freeze Dryer (Thermo, USA) and stored at − 80 °C. Quality control was carried out by a high-performance lipid chromatography (HPLC) using a 2695 liquid chromatography system (Waters, USA) as previously described [6].

### Experimental animals

Twenty male SD rats (7-week old, 200–250 g, SPF grade) were purchased from the Changchun Yisi laboratory animal technology Co, Ltd. (Changchun, China). The experimental animal certification number was SCXK (Ji) 2016-0003. The rats were housed in an air conditioned room (temperature 22 ± 2 °C, relative humidity 50 ± 10%) under a 12/12 h light/dark cycle. All experimental procedures were approved by the Institutional Animal Care and Use Committee of Changchun University of Chinese Medicine, and all experiments were performed in accordance with relevant guidelines and regulations.

### Drug administration

SD rats were randomly divided into two groups (10 rats per group). The rats in GZZSZTW group received oral administration at a dose of 1.05 g/kg once daily for 21 days and the rats in control group were fed with drinking water. The dose selected for GZZSZTW in animal experiment was calculated based on normalization to interspecies differences in body surface area [7].

### Tissue collection

Long bone and kidney were collected in the early morning after administration for 21 days. Briefly, one animal at a time was euthanized with CO_2_, and cervical dislocation was then performed to assure death. Tibiae and femurs were collected by removing muscles, tendons, ligaments, and any other irrelevant tissues and stored at − 80 °C for RNA extraction. The kidneys were collected from both sides of each rat and stored at − 80 °C for RNA extraction.

### RNA isolation and Illumina sequencing

Bone and kidney from each group were pooled together and ground into a fine powder respectively. In other words, we pooled the bones of the control group, bones of the group treated with GZZSZTW, kidneys of the control group, and kidneys of the group treated with GZZSZTW separately. Total RNA was extracted with the TRIzol reagent (Invitrogen, USA) according to the manufacturer’s protocol. RNA quality was determined by calculating RNA integrity number (RIN) using an Agilent 2100 Bioanalyzer (Agilent Technologies, USA). Paired-end mRNA libraries were prepared with the TruSeq Stranded mRNA kit (Illumina, USA) according to the manufacturer’s protocol. Transcriptome sequencing by RNA-seq was carried out on an Illumina HiSeq 2500 platform (Illumina, USA).

### RNA-seq data analysis

After RNA-seq, clean reads were obtained by filtering out the low-quality reads and adapter sequences. The clean reads from each sample were mapped to the rat (*Rattus norvegicus*) reference genome with HISAT [8]. Gene expression levels were calculated by the FPKM algorithm [9]. BLAST was used to perform annotations against the non-redundant (NR) and Swiss-Prot protein databases. Differentially expressed genes were identified according to an R package named DEGseq [10]. Genes with a log_2_ fold change ≥ 1 or ≤ − 1 and with a *p* value ≤ 0.001 were considered as differentially expressed genes.

### Function and pathway enrichment analysis of differentially expressed genes

Gene ontology (GO) and Kyoto Encyclopedia of Genes and Genomes (KEGG) enrichment analysis was carried out with R function phyper. The Hypergeometric test and Bonferroni correction were applied in the enrichment analysis. After multiple testing corrections, the GO terms or pathways with a corrected *p* value (Q value) less than 0.05 were considered significantly enriched in the differentially expressed genes [11].

### Quantitative real-time PCR validation of gene expression levels

qRT-PCR was used to validate the expression levels of differentially expressed genes identified by RNA-seq analysis. Briefly, total RNA was extracted with the TRIzol reagent (Invitrogen, USA) according to the manufacturer’s protocol. cDNA was synthesized using the iScript cDNA Synthesis Kit (Bio-Rad, USA) and amplified using SsoAdvanced Universal SYBR® Green Supermix (Bio-Rad, USA) on a CFX Connect Real-Time PCR Detection System (Bio-Rad, USA) under standard amplification conditions. The gene expression levels were normalized to the rat glyceraldehyde 3-phosphate dehydrogenase gene (*Gapdh*) and calculated using the 2^-ΔΔCT^ method [12].

## Results

### RNA-seq, transcriptome assembly, and functional annotation

The transcriptomes of bone and kidney from rats with or without the treatment of GZZSZTW were separately sequenced using paired-end Illumina sequencing method. All read sequences were deposited in the NCBI Sequence Read Archive (SRA) database under accession numbers SRP156418 and SRP139876. After removing low-quality reads and adapter sequences, 40,807,400 and 40,927,626 clean reads were obtained from the bone of rats, whereas 48,061,134 and 47,604,912 were obtained from the kidney of rats, which were not treated with GZZSZTW (blank) or treated with GZZSZTW, respectively, as shown in Table S1. The quality assessment showed that the Q30 percentages were greater than 92%, and the GC content percentages were approximately 50%. For the blank and GZZSZTW-treated bone samples, 33,335,432 and 31,169,550 reads were mapped to the rat genome, respectively. In total, 11,727 out of 14,418 (blank) and 11,584 out of 14,093 (GZZSZTW) transcripts were annotated against the non-redundant (NR) NCBI protein database and Swiss-Prot database, respectively. For the blank and GZZSZTW-treated kidney samples, 41,069,248 and 40,145,872 reads were mapped to the rat genome, respectively. In total, 12,802 out of 15,476 (blank) and 12,856 out of 15,563 (GZZSZTW) transcripts were annotated against the non-redundant (NR) NCBI protein database and Swiss-Prot database, respectively.

### Comparative analysis of differentially expressed genes

For the blank and GZZSZTW-treated bone samples, the differential expression analysis identified 2295 genes that were significantly differentially expressed between the GZZSZTW-treated and blank groups (log_2_ fold change ≥ 1 or ≤ − 1 and *p* ≤ 0.001), including 929 upregulated genes and 1366 downregulated genes (GZZSZTW vs. blank): For the blank and GZZSZTW-treated kidney samples, the differential expression analysis identified 709 genes that were significantly differentially expressed between the GZZSZTW-treated and blank groups (log_2_ fold change ≥ 1 or ≤ − 1 and *p* ≤ 0.001), including 255 upregulated genes and 454 downregulated genes (GZZSZTW vs. Blank), as shown in Table S2.

### GO and KEGG enrichment analysis of differentially expressed genes in bone and kidney under GZZSZTW treatment

**Fig. 1:**
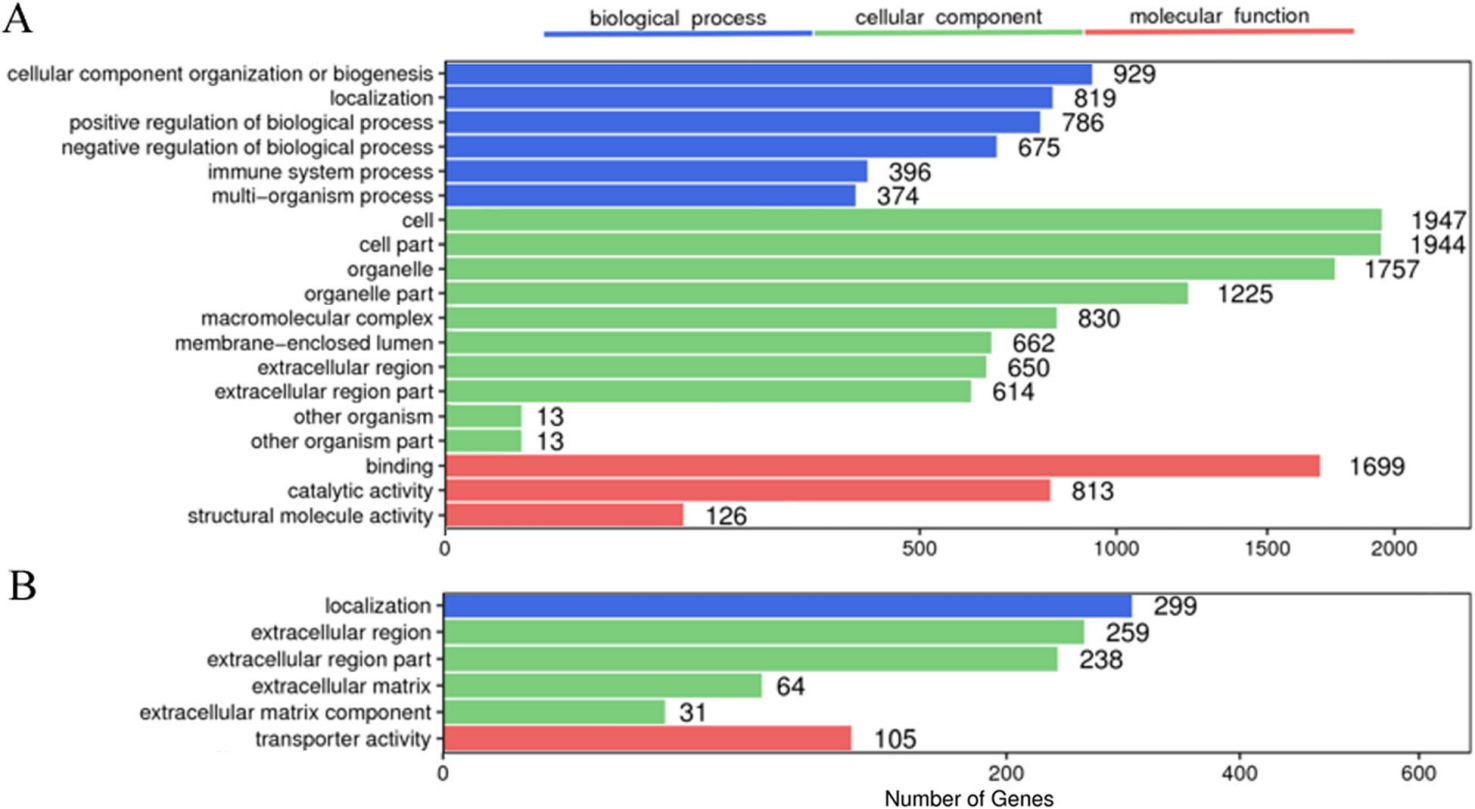
GO enrichment analysis of differentially expressed genes in bone and kidney under GZZSZTW treatment. **a** Bone. **b** Kidney. The x-axis indicates the number of genes in a category. The y-axis indicates the significantly (*p* < 0.05) enriched GO terms in the categories of biological process, molecular function, and cellular component

GO enrichment analyses were performed to gain insight into the differentially expressed genes involved in bone and kidney functions under GZZSZTW treatment, as shown in Fig. 1. For bone, the significantly enriched GO terms related with biological process were mainly involved in the categories of cellular component organization or biogenesis, localization, and regulation of biological process; the significantly enriched GO terms related with cellular component were mainly involved in the categories of cell part, organelle part, and macromolecular complex; the significantly enriched GO terms related with molecular function were mainly involved in the categories of binding, catalytic activity, and structural molecule activity. For the kidneys, the significantly enriched GO terms related with biological process were mainly involved in the categories of localization; the significantly enriched GO terms related with cellular component were mainly involved in the categories of extracellular region and extracellular matrix; the significantly enriched GO terms related with molecular function were mainly involved in the categories of transporter activity.

**Fig. 2:**
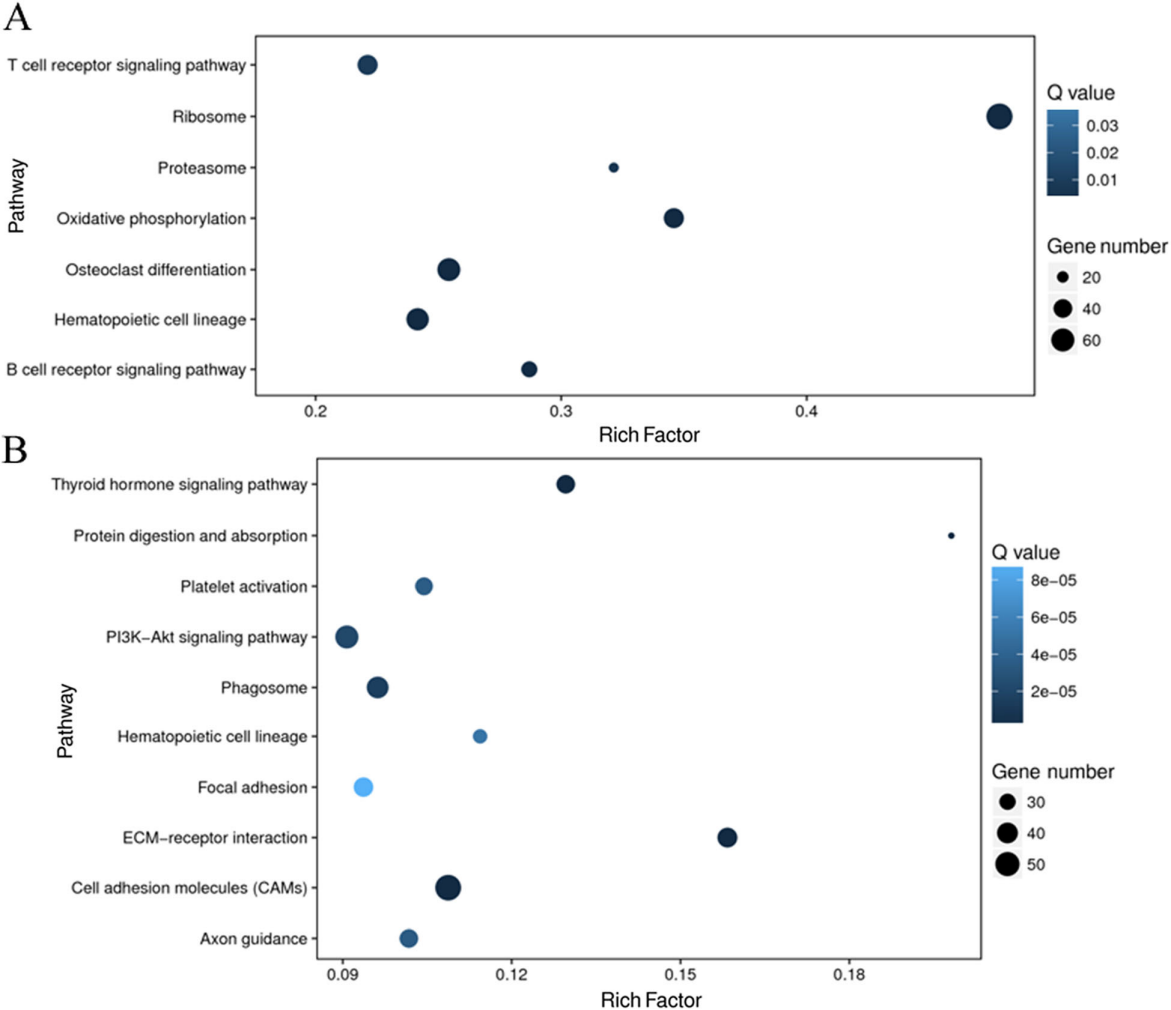
KEGG enrichment analysis of differentially expressed genes in bone and kidney under GZZSZTW treatment. **a** Bone. **b** Kidney. The x-axis represents rich factor, which is the ratio of the number of target genes divided by the number of all the genes in each pathway, and the y-axis represents the enriched pathway. The size and color of the dots represent the gene number and the range of Q values, respectively

KEGG pathway enrichment analyses were performed to further explore the possible physiological processes and pathways of these differentially expressed genes involved in bone and kidney functions under GZZSZTW treatment, as shown in Fig. 2. For the bone, the significant enriched pathways were mainly involved in the categories of ribosome, osteoclast differentiation, hematopoietic cell lineage, oxidative phosphorylation, T cell receptor signalling pathway, B cell receptor signalling pathway, and proteasome. For the kidney, the significant enriched pathways were mainly involved in the categories of cell adhesion molecules, PI3K-AKT signalling pathway, phagosome, ECM-receptor interaction, thyroid hormone signalling pathway, protein digestion and absorption, platelet activation, axon guidance, hematopoietic cell lineage, and focal adhesion.

### GZZSZTW decreases the expression levels of bone markers involved in bone formation and resorption

OA is a degenerative joint disease characterized by both progressive degeneration of articular cartilage and osteophyte formation. In our previous studies, GZZSZTW has been shown to promote chondrocyte proliferation and inhibit differentiation that would possibly contribute to cartilage regeneration in treating OA [4, 5]; therefore, we further argued whether GZZSZTW could also inhibit osteophyte formation in treating OA through suppressing bone cell markers that were involved in bone formation and resorption. As shown in Table 1, the expression levels of osteoblast, osteoclast, and osteophyte markers were significantly decreased in the bone under GZZSZTW treatment.

**Table 1:** The expression levels of bone cell markers (GZZSZTW vs. Blank)

Gene name	Blank (FPKM)	GZZSZTW (FPKM)	log_2_ fold change (GZZSZTW/Blank)	*p* value
Osteoblast markers				
Collagen alpha-1(I) chain (*Col1a1*)	198.95	66.96	− 1.57	0
Alkaline phosphatase, (*Alpl*)	15.81	5.33	− 1.57	6.62E−20
Bone sialoprotein 2 (*Ibsp*)	91.97	17.38	− 2.40	2.20E−150
Osteocalcin (*Bglap*)	872.04	147.20	− 2.57	6.10E−313
Osteopontin (*Spp1*)	96.37	34.42	− 1.49	5.87E−64
Osteomodulin (*Omd*)	7.34	0.75	− 3.29	1.52E−16
Decorin (*Dcn*)	56.87	19.21	− 1.57	2.30E−45
Osteoclast markers				
Tartrate-resistant acid phosphatase type 5 (*Acp5*)	103.22	13.98	− 2.88	6.00E−147
Cathepsin K (*Ctsk*)	82.80	6.46	− 3.68	3.00E−150
Nuclear factor of activated T-cells, cytoplasmic 3 (*Nfatc3*)	6.77	3.28	− 1.05	5.21E−12
Myc proto-oncogene protein (*Myc*)	14.37	6.47	− 1.15	1.13E−11
Osteocyte markers				
Podoplanin (*Pdpn*)	3.33	1.10	− 1.60	8.21E−04
Dentin matrix acidic phosphoprotein 1 (*Dmp1*)	45.75	5.78	− 2.98	7.15E−132
Sclerostin (*Sost*)	11.75	0.57	− 4.37	2.85E−11
Metalloendopeptidase homolog PEX (*Phex*)	0.82	0.12	− 2.77	9.62E−05
Matrix extracellular phosphoglycoprotein (*Mepe*)	40.91	4.96	− 3.04	2.53E−73
Dickkopf-related protein 1 (*Dkk1*)	3.65	0.37	− 3.30	3.92E−11

### GZZSZTW increases the expression levels of kidney markers and regulators involved in kidney function, regulation, and repair

According to the “kidney governing bone” theory of traditional Chinese medicine, we hypothesized that the effect of GZZSZTW on treating OA may be achieved by the regulation of kidney function. Our results showed that GZZSZTW indeed increased the expression levels of kidney markers and regulators involved in kidney function, regulation and repair, although the expression levels of the majority of these genes were only slightly increased under GZZSZTW treatment, as shown in Table 2.

**Table 2:** The expression levels of kidney markers and regulators involved in kidney function, regulation, and repair (GZZSZTW vs. Blank)

Gene name	Blank (FPKM)	GZZSZTW (FPKM)	log_2_ fold change (GZZSZTW/Blank)	*p* value
Kidney markers				
Uromodulin (*Umod*)	1882.20	3124.23	0.73	0
Solute carrier family 7 member 13 (*Slc7a13*)	947.10	1462.48	0.63	0
Solute carrier family 12 member 1 (*Slc12a1*)	344.12	554.10	0.69	0
Aquaporin-2 (*Aqp2*)	325.29	485.70	0.58	7.21E−88
*Solute carrier family 22 member 12 (*Slc22a12*)	122.60	279.04	1.19	2.06E−266
Transmembrane protein 52B (*Tmem52b*)	138.18	239.13	0.79	1.26E−107
Solute carrier family 12 member 3 (*Slc12a3*)	121.71	192.57	0.66	6.73E−128
Transmembrane protein 174 (*Tmem174*)	124.35	143.94	0.21	1.05E−05
N-acetyltransferase 8 (*Nat8*)	114.50	115.27	0.01	5.98E−01
FXYD domain-containing ion transport regulator 4 (*Fxyd4*)	83.00	114.50	0.46	1.31E−08
Aquaporin-6 (*Aqp6*)	45.56	88.40	0.96	3.48E−36
*Solute carrier family 22 member 13 (*Slc22a13*)	29.45	62.88	1.09	2.27E−79
Transmembrane protein 72 (*Tmem72*)	41.21	55.59	0.43	1.39E−25
Podocin (*Nphs2*)	43.01	52.46	0.29	1.37E−04
Pendrin (*Slc26a4*)	26.32	51.83	0.98	1.20E−49
Nephrin (*Nphs1*)	18.71	32.02	0.78	1.87E−43
Chloride channel protein ClC-Ka (*Clcnka*)	19.56	31.06	0.67	1.00E−14
Paired box protein Pax-2 (*Pax2*)	17.22	19.31	0.17	3.71E−02
Single-minded homolog 1 (*Sim1*)	9.44	11.14	0.24	6.44E−02
Transient receptor potential cation channel subfamily V member 5 (*Trpv5*)	2.62	4.00	0.61	2.98E−04
Uroplakin-1a (*Upk1a*)	2.26	3.75	0.73	3.43E−02
*Sclerostin (*Sost*)	0.01	3.06	8.26	3.22E−05
Kidney regulator				
*Protein S100-A6 (*S100a6*)	85.59	204.01	1.25	3.25E−29
*Clusterin (*Clu*)	38.41	87.28	1.18	1.38E−66
*Vimentin (*Vim*)	29.83	67.37	1.18	4.97E−53
*Solute carrier family 22 member 13 (*Slc22a13*)	29.45	62.88	1.09	2.27E−79
*Dexamethasone-induced Ras-related protein 1 (*Rasd1*)	3.14	6.68	1.09	2.22E−05
Annexin A1 (*Anxa1*)	66.96	105.55	0.66	3.53E−25
Neutrophil gelatinase-associated lipocalin (*Lcn2*)	6.19	10.56	0.77	3.93E−03
Actin-related protein 2/3 complex subunit 1B (*Arpc1b*)	61.49	74.90	0.28	1.22E−05
Cyclic AMP-dependent transcription factor ATF-3 (*Atf3*)	1.51	1.72	0.19	6.07E−01
Claudin-4 (*Cldn4*)	20.40	32.96	0.69	3.13E−12
Metalloproteinase inhibitor 1 (*Timp1*)	3.05	5.24	0.78	7.42E−02
Annexin A2 (*Anxa2*)	62.35	71.45	0.20	3.37E−03
Signal transducer CD24 (*Cd24*)	154.61	215.25	0.48	8.30E−39
Prominin-1 (*Prom1*)	12.64	18.60	0.56	1.18E−10
Arginase-1 (*Arg1*)	0.93	1.98	1.09	3.03E−02
Protein Wnt-7b (*Wnt7b*)	7.57	10.73	0.50	1.36E−02
Macrophage colony-stimulating factor 1 (*Csf1*)	4.12	6.26	0.60	2.88E−01
Fibrinogen beta chain (*Fgb*)	5.70	6.81	0.26	2.38E−01
Fibrinogen alpha chain (*Fga*)	13.81	14.93	0.11	3.63E−01

*significantly upregulated genes under GZZSZTW treatment

### GZZSZTW treatment inhibits osteophyte formation by regulating multiple local genes that regulate bone homeostasis

In order to explore the molecular mechanisms underlying osteophyte inhibition under GZZSZTW treatment, we further analyzed the expression levels of multiple local genes that positively regulate bone homeostasis. As shown in Table 3, the expression levels of *Pth1r*, *Tgfb1*, *Bmp4*, *Bmp1*, *Fgfr3*, *Dlx3*, *Nfatc3*, and several subtypes of protein S100 were significantly increased under GZZSZTW treatment.

**Table 3:** The expression levels of local genes that regulate bone homeostasis (GZZSZTW vs. Blank)

Gene name	Blank (FPKM)	GZZSZTW (FPKM)	log_2_ fold change (GZZSZTW /Blank)	*p* value
Parathyroid hormone/parathyroid hormone-related peptide receptor (*Pth1r*)	13.93	3.61	− 1.95	7.50E−18
Transforming growth factor beta-1 (*Tgfb1*)	42.33	18.87	− 1.17	3.14E−20
Bone morphogenetic protein 4 (*Bmp4*)	4.75	1.32	− 1.85	1.09E−05
Bone morphogenetic protein 1 (*Bmp1*)	4.19	1.85	− 1.18	1.75E−06
Fibroblast growth factor receptor 3 (*Fgfr3*)	0.88	0.05	− 4.14	5.45E−05
Leptin receptor (*Lepr*)	4.28	2.11	− 1.02	3.39E−06
Homeobox protein DLX-3 (*Dlx3*)	1.97	0.25	− 2.98	8.54E−07
Nuclear factor of activated T-cells, cytoplasmic 3 (*Nfatc3*)	6.77	3.28	− 1.05	5.21E−12
Protein S100-A4 (*S100a4*)	1438.55	401.73	− 1.84	0
Protein S100-A6 (*S100a6*)	470.14	179.89	− 1.39	9.97E−61
Protein S100-A10 (*S100a10*)	267.50	104.41	− 1.36	5.29E−52

### Kidney governing bone through multiple functional genes involved in both kidney and bone homeostasis

In order to discover the scientific connotation of the “kidney governing bone” theory, we screened the differentially expressed genes of which the expression levels were significantly changed in both the kidney and bone under GZZSZTW treatment. As shown in Table 4, the expression levels of 15 genes were significantly increased in both the kidney and bone, such as *Catsper2*, *Per3*, *Slfn12l*, *Hmha1*, and *Junb.* As shown in Table 5, the expression levels of 39 genes were significantly decreased in both the kidney and bone, such as *Trim59*, *Col6a2*, *Loxl1*, *Rt1ba*, and *Mmp2*.

**Table 4:** Significantly upregulated genes in both bone and kidney (GZZSZTW vs. Blank)

Gene name	Bone				Kidney			
	Blank (FPKM)	GZZSZTW (FPKM)	log_2_ fold change (GZZSZTW/Blank)	*p* value	Blank (FPKM)	GZZSZTW (FPKM)	log_2_ fold change (GZZSZTW/Blank)	*p* value
Cation channel sperm-associated protein 2 (*Catsper2*)	0.51	2.63	2.37	2.02E−06	1.68	3.96	1.24	2.67E−05
Period circadian protein homolog 3 (*Per3*)	1.39	4.17	1.58	3.29E−05	2.19	6.66	1.60	4.74E−18
Schlafen family member 12-like (*Slfn12l*)	38.51	264.68	2.78	0	1.29	2.62	1.02	4.83E−06
Minor histocompatibility protein HA-1 (*Hmha1*)	26.16	74.34	1.51	4.20E−138	1.40	2.93	1.07	9.10E−06
Transcription factor jun-B (*Junb*)	13.65	36.86	1.43	4.01E−30	18.24	51.84	1.51	3.68E−60
Cadherin-23 (*Cdh23*)	0.19	0.83	2.13	1.45E−08	0.09	0.71	2.98	9.60E−14
Fetuin-B (*Fetub*)	0.93	4.99	2.42	1.73E−07	2.63	7.66	1.54	6.98E−10
Protocadherin gamma-A11 (*Pcdhga11*)	0.01	0.48	5.58	3.61E−05	0.10	0.70	2.81	6.73E−06
Protein BANP (*Banp*)	4.52	13.57	1.59	1.40E−18	3.91	9.66	1.30	5.39E−15
Protein phosphatase 1 regulatory subunit 1A (*Ppp1r1a*)	0.46	2.52	2.45	9.74E−05	174.26	401.40	1.20	1.34E−274
Beta-enolase (*Eno3*)	5.72	32.64	2.51	4.42E−44	1.82	4.82	1.41	1.12E−04
Ankyrin repeat domain-containing protein 13D (*Ankrd13d*)	1.40	3.55	1.34	2.71E−04	0.43	1.71	1.99	1.22E−04
Autophagy-related protein 16-2 (*Atg16l2*)	4.13	9.46	1.20	3.16E−08	1.05	2.71	1.37	1.85E−04
B cell lymphoma 6 protein homolog (*Bcl6*)	3.50	8.67	1.31	9.91E−13	3.06	6.38	1.06	1.39E−09
Proto-oncogene c-Fos (*Fos*)	5.47	12.82	1.23	2.31E−12	0.75	10.37	3.79	7.87E−31

**Table 5:** Significantly down-regulated genes in both bone and kidney (GZZSZTW vs. Blank)

Gene name	Bone				Kidney			
	Blank (FPKM)	GZZSZTW (FPKM)	log_2_ fold change (GZZSZTW/Blank)	*p* value	Blank (FPKM)	GZZSZTW (FPKM)	log_2_ fold change (GZZSZTW/Blank)	*p* value
Tripartite motif-containing protein 59 (*Trim59*)	63.96	30.75	− 1.06	4.41E−47	3.76	0.91	− 2.05	2.92E−11
Collagen alpha-2(VI) chain (*Col6a2*)	3.99	1.59	− 1.33	5.97E−07	6.90	1.73	− 2.00	3.15E−25
Lysyl oxidase homolog 1 (*Loxl1*)	2.55	0.87	− 1.55	2.63E−04	6.67	3.20	− 1.06	2.23E−07
Rano class II histocompatibility antigen, B alpha chain (*Rt1ba*)	16.32	4.02	− 2.02	1.59E−13	83.32	40.73	− 1.03	1.32E−30
72 kDa type IV collagenase (*Mmp2*)	4.72	1.68	− 1.49	6.19E−08	4.49	1.63	− 1.46	1.11E−09
Collagen alpha-1(V) chain (*Col5a1*)	1.84	0.38	− 2.28	1.27E−09	3.71	1.35	− 1.46	3.82E−21
Olfactomedin-like protein 3 (*Olfml3*)	6.31	0.48	− 3.72	2.17E−15	7.82	2.56	− 1.61	1.51E−10
Mono [ADP-ribose] polymerase PARP16 (*Parp16*)	4.18	1.65	− 1.34	4.41E−05	16.13	6.90	− 1.23	1.45E−20
Hemoglobin subunit beta-1 (*Hbb1*)	318449.91	105658.11	− 1.59	0	1173.62	298.69	− 1.97	0
Aryl hydrocarbon receptor nuclear translocator-like protein 1 (*Arntl*)	1.55	0.06	− 4.69	2.94E−08	12.17	1.14	− 3.42	2.01E−60
Apolipoprotein B mRNA editing enzyme, catalytic polypeptide-like 3A (*Apobec3a*)	0.76	0.17	− 2.16	1.95E−04	2.11	1.00	− 1.08	4.95E−06
Hemoglobin subunit beta-2 (*Hbb2*)	25744.68	10685.16	− 1.27	0	107.40	24.59	− 2.13	2.24E−56
Galectin-5 (*Lgals5*)	619.97	306.05	− 1.02	2.49E−117	5.43	1.47	− 1.89	8.97E−05
Mitochondrial fission regulator 1 (*Mtfr1*)	9.27	2.99	− 1.63	4.01E−06	22.05	10.25	− 1.11	1.90E−09
Solute carrier organic anion transporter family member 2B1 (*Slco2b1*)	3.18	1.00	− 1.67	6.28E−08	3.65	1.07	− 1.77	3.73E−13
Macrophage-expressed gene 1 protein (*Mpeg1*)	22.34	9.15	− 1.29	1.29E−32	4.09	1.94	− 1.08	5.11E−08
Insulin-like growth factor-binding protein 4 (*Igfbp4*)	22.16	10.42	− 1.09	7.73E−15	80.24	20.72	− 1.95	6.94E−157
Decorin (*Dcn*)	56.87	19.21	− 1.57	2.30E−45	87.18	20.12	− 2.12	4.97E−145
Calcipressin-1 (*Rcan1*)	6.83	3.03	− 1.17	5.07E−06	46.91	23.12	− 1.02	1.38E−33
Phosphoserine aminotransferase (*Psat1*)	6.86	2.84	− 1.27	2.21E−06	191.68	64.45	− 1.57	9.36E−259
Selenoprotein P (*Sepp1*)	118.47	23.76	− 2.32	1.56E−194	1943.71	875.64	− 1.15	0
Coronin-1C (*Coro1c*)	4.42	0.61	− 2.86	1.71E−07	13.29	5.98	− 1.15	1.06E−08
Collagen alpha-1(I) chain (*Col1a1*)	198.95	66.96	− 1.57	0	7.51	1.09	− 2.78	1.51E−64
DNA damage-inducible transcript 4 protein (*Ddit4*)	7.25	2.05	− 1.82	7.97E−09	43.56	21.42	− 1.02	3.31E−26
Endoglin (*Eng*)	3.61	0.85	− 2.09	6.83E−09	36.46	17.26	− 1.08	4.64E−41
Monocarboxylate transporter 1 (*Slc16a1*)	40.94	18.51	− 1.15	2.32E−32	56.75	5.62	− 3.34	1.52E−236
Slit homolog 3 protein (*Slit3*)	1.32	0.28	− 2.24	8.69E−07	2.19	1.02	− 1.10	6.17E−06
Hephaestin (*Heph*)	2.31	0.85	− 1.44	1.15E−05	3.50	1.56	− 1.17	8.10E−09
Caspase-4 (*Casp4*)	6.86	2.71	− 1.34	9.64E−05	5.85	2.47	− 1.24	9.73E−05
Carboxypeptidase Q (*Cpq*)	7.40	1.89	− 1.97	1.29E−09	136.94	65.28	− 1.07	3.24E−93
Aurora kinase B (*Aurkb*)	14.57	6.47	− 1.17	1.52E−09	0.77	0.01	− 6.27	2.34E−04
Complement C1q tumor necrosis factor-related protein 6 (*C1qtnf6*)	2.76	0.50	− 2.46	5.54E−07	1.31	0.29	− 2.18	1.81E−04
Glia-derived nexin (*Serpine2*)	85.20	34.85	− 1.29	1.67E−60	8.47	1.53	− 2.47	1.82E−22
A disintegrin and metalloproteinase with thrombospondin motifs 2 (*Adamts2*)	1.28	0.27	− 2.25	5.20E−08	1.02	0.26	− 1.97	3.48E−05
Glutathione peroxidase 3 (*Gpx3*)	186.91	71.69	− 1.38	5.73E−109	12320.88	4891.04	− 1.33	0
Arachidonate 15-lipoxygenase (*Alox15*)	34.62	13.20	− 1.39	2.33E−32	1.61	0.10	− 4.01	6.27E−08
Insulin-like growth factor-binding protein 3 (*Igfbp3*)	5.44	2.70	− 1.01	1.45E−04	64.08	16.86	− 1.93	9.00E−137
Collagen alpha-1(III) (*Col3a1*)	18.19	4.02	− 2.18	3.35E−68	17.97	2.29	− 2.97	6.13E−133
Osteomodulin (*Omd*)	7.34	0.75	− 3.29	1.52E−16	3.64	1.41	− 1.37	9.08E−05

### qRT-PCR Validation of RNA-seq data

**Fig. 3:**
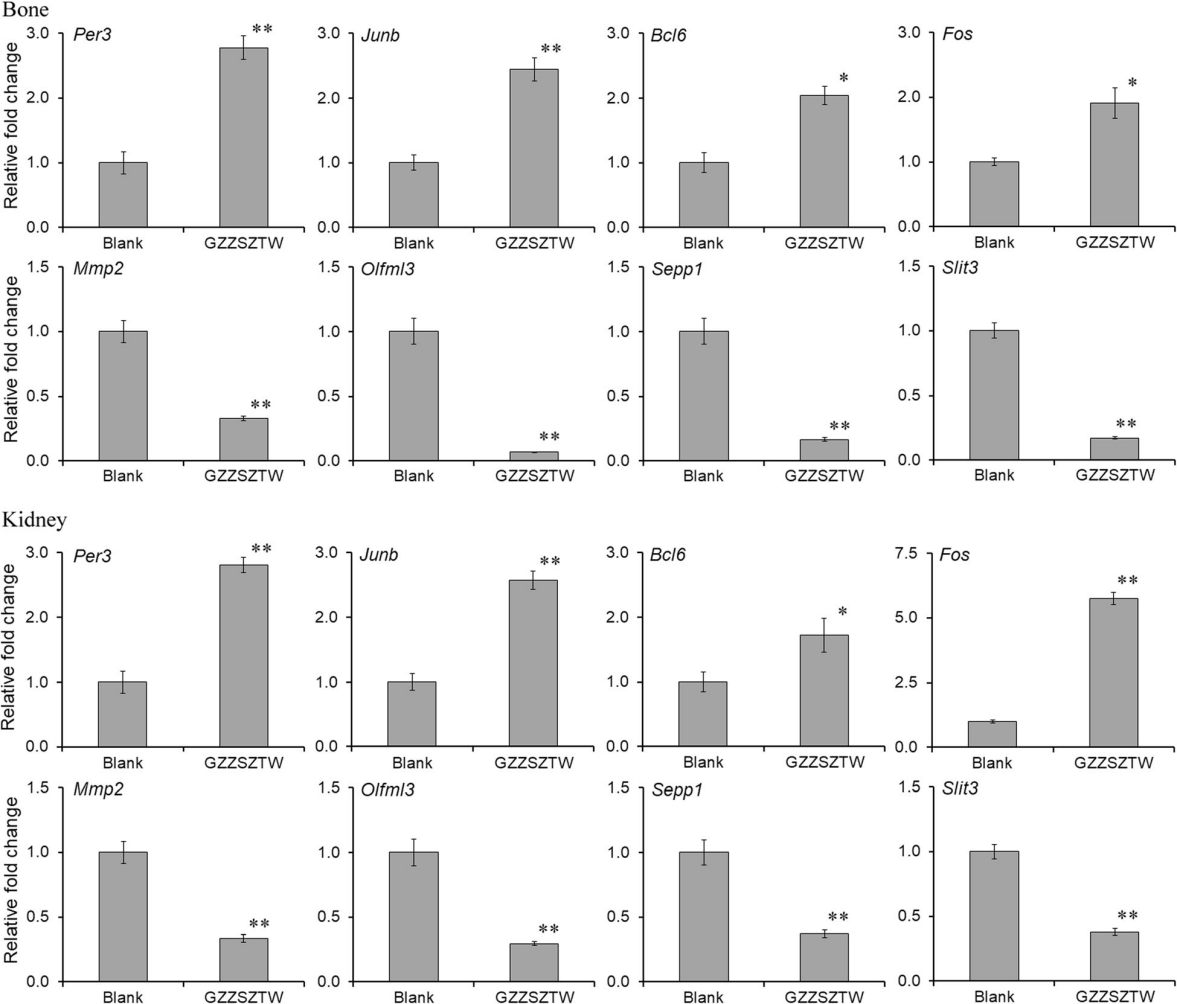
Gene expression levels of differentially expressed genes validated by qRT-PCR. Data are presented as the mean with standard deviation for technical triplicates in an experiment representative of several independent ones. * represents *p* < 0.01 and ** represents *p* < 0.001 in a *t* test for the difference in gene expression level. Gene expression levels for individual genes are presented as the fold change between the GZZSZTW-treated group (GZZSZTW) and the untreated group (Blank)

We validated the expression levels of 8 differentially expressed genes by qRT-PCR method, including 4 significantly upregulated genes (*Per3*, *Junb*, *Bcl6*, and *Fos*) and 4 significantly downregulated genes (*Mmp2*, *Olfml3*, *Sepp1*, and *Slit3*) which were significantly changed in both the bone and kidney. The specific primers used in this experiment are listed in Table S3. The relative fold change of each gene was normalized to the internal reference gene *Gapdh*. The expression levels of the selected differentially expressed genes measured by qRT-PCR were consistent with the results of the RNA-seq analysis, as shown in Fig. 3.

## Discussion

“Kidney governing bone” is a fundamental theory in traditional Chinese medicine. This theory is used as a gold guide for the treatment of bone diseases in traditional Chinese medicine. However, the current studies mainly focus on its applications in treating osteoporosis, whereas little is known about its role in the treatment of cartilage diseases, such as OA [13]. GZZSZTW is a classic Chinese medicinal formula that has been widely used for treating joint diseases. GZZSZTW has been shown to stimulate chondrocyte proliferation, prevent chondrocyte differentiation, and modulate chondrocyte structure, dynamics, and metabolism by controlling multiple functional genes and proteins [4, 5]. However, the precise molecular mechanism of GZZSZTW in terms of the “kidney governing bone” theory to treat cartilage diseases remains to be elucidated. In the present study, we investigated the effects of GZZSZTW on both the kidney and bone using a state-of-the-art RNA-seq technology.

In total, 2295 differentially expressed genes were identified in bone under the GZZSZTW treatment, including 929 upregulated genes and 1366 downregulated genes (GZZSZTW vs. Blank). Seven hundred nine differentially expressed genes were identified in the kidney under the GZZSZTW treatment, including 255 upregulated genes and 454 downregulated genes (GZZSZTW vs. Blank). Based on the GO enrichment analysis, the significantly enriched GO terms for the bone transcriptome were mainly involved in the categories of binding, catalytic activity, and structural molecule activity, whereas the significantly enriched GO terms for the kidney transcriptome were mainly involved in the categories of transporter activity. According to the KEGG enrichment analysis, the significant enriched pathways of the bone transcriptome were mainly involved in the categories of ribosome, osteoclast differentiation, hematopoietic cell lineage, oxidative phosphorylation, T cell receptor signalling pathway, B cell receptor signalling pathway, and proteasome. The significant enriched pathways of the kidney transcriptome were mainly involved in the categories of cell adhesion molecules, PI3K-AKT signalling pathway, phagosome, ECM-receptor interaction, thyroid hormone signalling pathway, protein digestion and absorption, platelet activation, axon guidance, hematopoietic cell lineage, and focal adhesion. These results indicated that GZZSZTW controlled the bone and kidney by modulating multiple functional genes and signalling pathways.

We first analyzed the bone and kidney markers involved in bone and kidney homeostasis. In the bone, the expression levels of osteoblast, osteoclast, and osteophyte markers were significantly decreased under GZZSZTW treatment. These results suggested that GZZSZTW significantly inhibited the processes of bone formation and resorption, which in turn reflected that GZZSZTW might play an important role in treating OA by preventing osteophyte formation. Osteophyte, a common feature of OA, is generated by the dysregulation of bone formation and resorption. Therefore, inhibition of osteophyte formation by inhibiting bone formation and resorption has been considered as an optional way to treat OA [14].

We then analyzed the expression levels of kidney markers and regulators involved in kidney regulation. Our results showed that GZZSZTW indeed increased the expression levels of kidney markers and regulators involved in kidney function, regulation, and repair, although the expression levels of a majority of these genes were only slightly increased under GZZSZTW treatment. These results suggested that the effect of GZZSZTW on treating OA might be achieved through the enhancement of kidney function. It is noteworthy that the expression levels of 7 kidney markers and regulators including *Slc22a12*, *Slc22a13*, *Sost*, *S100a6*, *Clu*, *Vim*, and *Rasd1* were significantly increased in the kidney under GZZSZTW treatment. *Slc22a12* and *Slc22a13* are two types of the solute carrier (*Slc*) family 22 transporters, which facilitate the transport of a number of substrates across biological membranes, including endogenous compounds, drugs, and herbal/dietary supplements [15]. *Sost* is a circulating protein with a capacity to inhibit bone formation through negatively regulating the Wnt/β-catenin signalling pathway [16]. *S100a6* is a calcium-binding protein that plays a crucial role in the regulation of renal cell proliferation and regeneration in the kidney recovery process [17]. *Clu* is a chaperone protein that is required for renal tissue regeneration during the process of kidney repair, which is associated with promotion of tubular cell proliferation [18]. *Vim* is a mesenchymal marker that is expressed in kidney interstitium during the process of renal regeneration [19]. *Rasd1*, a dexamethasone-inducible Ras protein, is predominantly expressed in the proximal tubules in the renal cortex and outer medulla, which are the nephron sites that play a crucial role in regulating the recovery process of kidney injury [20]. Therefore, our results indicated that the effect of GZZSZTW on inhibiting osteophyte formation during the process of OA might be achieved by enhancing kidney function and then suppressing bone formation and resorption, which were guided by the “kidneys governing bone” theory.

Next, we analyzed the expression levels of local genes in the bone that regulate bone homeostasis, including *Pth1r*, *Tgfb1*, *Bmp4*, *Bmp1*, *Fgfr3*, *Lepr*, *Dlx3*, *Nfatc3*, *S100a4*, *S100a6*, and *S100a10*. Our results showed that the expression levels of all these local genes were significantly increased under GZZSZTW treatment. *Pth1r*, a G protein-coupled parathyroid hormone 1 receptor, is highly expressed in kidney and bone with a distinct role in modulating systemic mineral ion homeostasis by maintaining calcium-phosphate balance via the cross-talk between the bone and kidney [21]. *Tgfb1*, *Bmp4*, and *Bmp1* belong to the transforming growth factor beta superfamily, which has fundamental roles in both embryonic skeletal development and postnatal bone homeostasis [22]. *Fgfr3*, a member of the fibroblast growth factor receptor family, plays a critical role in the control of endochondral ossification and bone growth [23]. *Lepr* is the receptor of leptin, which acts as an important regulator on bone growth and metabolism [24]. *Dlx3* is a homeodomain-containing transcription factor that is involve in the regulation of bone formation and homeostasis in the adult skeleton [25]. *Nfatc3*, a family member of the nuclear factors of activated T cells, plays an important role in regulating bone formation and resorption through the receptor activator of NF-κB ligand (RANKL) [26]. *S100a4*, *S100a6*, and *S100a10*, which belong to the calcium-binding S100 family members, have been shown to regulate bone formation and resorption [27–29]. Therefore, these results were consistent with the above findings that the effect of GZZSZTW on inhibiting osteophyte formation during the process of OA might be achieved by enhancing kidney function and then suppressing bone formation and resorption.

Finally, we analyzed the differentially expressed genes with consistent patterns in both the kidney and bone, of which 15 genes were significantly upregulated and 39 genes were significantly downregulated in both the kidney and bone under GZZSZTW. Among the upregulated genes in both the kidney and bone, *Per3*, *Junb*, *Bcl6*, and *Fos* have been reported to be involved in the regulation of bone formation and resorption by controlling osteoblast and osteoclast activities [30–33]. Among the downregulated genes in both the kidney and bone, *Col6a2*, *Mmp2*, *Col5a1*, *Olfml3*, *Igfbp4*, *Dcn*, *Sepp1*, *Col1a1*, *Slc16a1*, *Slit3*, *Alox15*, *Igfbp3*, *Col3a1*, and *Omd* have been reported to be involved in the regulation of bone formation and resorption by controlling osteoblast and osteoclast activities [34–47]. Therefore, these results suggest that the effects of GZZSZTW might be achieved by modulating the activities of osteoblast and osteoclast via the modulation of the kidney on the bone according to the “kidney governing bone” theory.

According to our previously published papers, we demonstrate that GZZSZTW stimulates chondrocyte proliferation, prevent chondrocyte differentiation, and modulate chondrocyte structure, dynamics, and metabolism by controlling multiple functional genes, proteins, and signalling pathways responsible for cartilage development, growth, and repair [4, 5]. Those results suggest that the clinical application of GZZSZTW in treating OA might be achieved partially by modulating chondrocyte proliferation and differentiation. In the present study, we further demonstrate that GZZSZTW might also play roles in regulating kidney and bone functions by modulating functional gene expression patterns, which might be related to the “kidney governing bone” theory that has been widely used as a golden guide in TCM for treating bone and cartilage diseases in clinic for many centuries. Taken together, we have currently discovered some clues regarding the molecular mechanisms of GZZSZTW in regulating cartilage and bone development, which might be governed by kidney function. However, other in vitro and in vivo approaches still need to be well designed and performed, such as proteomic analysis, histological analysis as well as gain- and loss-of-function analyses, to fully dissect the underlying mechanisms of GZZSZTW on bone and kidney regulation based on the “kidney governing bone” theory.

Overall, this is the first attempt to investigate the potential pharmaceutical mechanism of GZZSZTW on the bone and kidney based on the “kidney governing bone” theory. Although this formulation has been used as an in-hospital preparation for several decades to treat joint diseases, it still could not become a national or even a global treatment option due to a lack of scientifically literate voices. The present study has deepened the current knowledge about the molecular effects of GZZSZTW on bone and kidney regulation. Furthermore, this study might be able to provide possible strategies to further prevent and treat joint diseases by using traditional Chinese medicinal formulations following the “kidney governing bone” theory. Thus, the present study will give a scientific support for the clinical use of GZZSZTW alone or in combination with other medications. However, although the present study has showed the mentioned clinical significance and advantages, it still shows some disadvantages. In this study, we only investigated the potential pharmaceutical mechanism of GZZSZTW on bone and kidney under normal physiological conditions, further study still needs to be carried out to investigate the effects of GZZSZTW on kidney and bone under pathological conditions, such as OA, and to fully address the pharmaceutical mechanism of GZZSZTW on treating joint diseases.

## Conclusion

In summary, the present study demonstrated that the Chinese medicinal formulation GZZSZTW, which has been used for several decades to treat joint diseases such as OA, could enhance kidney function and suppress bone formation and resorption by modulating the activities of osteoblast and osteoclast, and might subsequently contribute to the inhibition of osteophyte formation during the process of OA. These effects might be achieved by the synergistic interactions of various herbs and their active components in GZZSZTW, which increased the expression levels of functional genes participating in kidney function, regulation, and repair, and then decreased the expression levels of genes involved in bone formation and resorption. Thus, our findings were consistent with the “kidney governing bone” theory, which has been widely used as a guide in clinical practice for thousands of years. Furthermore, this study might be able to provide possible strategies to further prevent and treat joint diseases by using traditional Chinese medicinal formulations following the “kidney governing bone” theory.

## Supplementary information

**Additional file 1: Table S1.** Statistics for the sequencing and assembly results

**Additional file 2: Table S2** Statistical analysis of differentially expressed genes in bone and kidney (GZZSZTW vs. Blank)

**Additional file 3: Table S3** Primer sequences for qRT-PCR validation

## Data Availability

The datasets used and/or analyzed during the current study are available from the corresponding author on reasonable request.

## Abbreviations

GZZSZTW: Guzhi Zengsheng Zhitongwan
RNA-seq: RNA sequencing
OA: Osteoarthritis
TCM: Traditional Chinese medicine
GO: Gene ontology
KEGG: Kyoto Encyclopedia of Genes and Genomes
qRT-PCR: Quantitative real-time PCR
Gapdh: Glyceraldehyde 3-phosphate dehydrogenase
SRA: Sequence Read Archive
