# Supplementary material for: Deciphering the potential pharmaceutical mechanism of Guzhi Zengsheng Zhitongwan on rat bone and kidney based on the “kidney governing bone” theory
Source: J Orthop Surg Res. 2020 Apr 15;15:146. doi: 10.1186/s13018-020-01677-8 (PMC7161198; doi:10.1186/s13018-020-01677-8)
Supplement: Supplementary file 1 — Additional file 1: Table S1. Statistics for the sequencing and assembly results [file 13018_2020_1677_MOESM1_ESM.doc]

**Table S1 Statistics for the sequencing and assembly results**

|  | Bone | | Kidney | |
| --- | --- | --- | --- | --- |
| Statistics | Blank | GZZSZTW | Blank | GZZSZTW |
| Clean reads | 40,807,400 | 40,927,626 | 48,061,134 | 47,604,912 |
| Q30 percentage | 93.94 | 92.30 | 93.14 | 93.32 |
| GC percentage | 50.03 | 45.13 | 50.13 | 49.60 |
| Total mapped reads | 33,335,432 | 31,169,550 | 41,069,248 | 40,145,872 |
| Total transcripts | 14,418 | 14,093 | 15,476 | 15,563 |
| Known transcripts | 11,727 | 11,584 | 12,802 | 12,856 |
