# Supplementary material for: Deciphering the potential pharmaceutical mechanism of Guzhi Zengsheng Zhitongwan on rat bone and kidney based on the “kidney governing bone” theory
Source: J Orthop Surg Res. 2020 Apr 15;15:146. doi: 10.1186/s13018-020-01677-8 (PMC7161198; doi:10.1186/s13018-020-01677-8)
Supplement: Supplementary file 2 — Additional file 2: Table S2 Statistical analysis of differentially expressed genes in bone and kidney (GZZSZTW vs. Blank) [file 13018_2020_1677_MOESM2_ESM.doc]

**Table S2 Statistical analysis of differentially expressed genes in bone and kidney (GZZSZTW vs. Blank)**

| Statistics | Number |
| --- | --- |
| *Bone* |  |
| Differentially expressed mRNAs | 2,295 |
| Up regulated mRNAs | 929 |
| Down regulated mRNAs | 1366 |
| *Kidney* |  |
| Differentially expressed mRNAs | 709 |
| Up regulated mRNAs | 255 |
| Down regulated mRNAs | 454 |
