# Supplementary material for: Deciphering the potential pharmaceutical mechanism of Guzhi Zengsheng Zhitongwan on rat bone and kidney based on the “kidney governing bone” theory
Source: J Orthop Surg Res. 2020 Apr 15;15:146. doi: 10.1186/s13018-020-01677-8 (PMC7161198; doi:10.1186/s13018-020-01677-8)
Supplement: Supplementary file 3 — Additional file 3: Table S3 Primer sequences for qRT-PCR validation [file 13018_2020_1677_MOESM3_ESM.doc]

**Table S3 Primer sequences for qRT-PCR validation**

| Gene | Primer | Sequence |
| --- | --- | --- |
| *Per3* | Forward primer | CCTGAGGAAAGACAGCTCCA |
|  | Reverse primer | AGGGTGGGACATCTTCACAG |
| *Junb* | Forward primer | CCATCAGCTACCTCCCACAT |
|  | Reverse primer | CCTCTTTAAAGGCGGAAGCG |
| *Bcl6* | Forward primer | AAGGCATTGGGCAAACACAA |
|  | Reverse primer | ATGCTGTAGAACAGGCCACT |
| *Fos* | Forward primer | AACGGAGAATCCGAAGGGAA |
|  | Reverse primer | GTTGATCTGTCTCCGCTTGG |
| *Mmp2* | Forward primer | GGTTACACACCTGACCTGGA |
|  | Reverse primer | TCCATCTCCATGCTCCCATC |
| *Olfml3* | Forward primer | AGGGCCGGAGAAATGAGAAA |
|  | Reverse primer | ACCAAACCGCTTCAGGATCT |
| *Sepp1* | Forward primer | ATGACAGATGTGGCCGTCTT |
|  | Reverse primer | CTCTGAGGGCTCTGTGGTTT |
| *Slit3* | Forward primer | TTCAACGGGCTAAGGTCACT |
|  | Reverse primer | GTGGAGAGGGTTGATTCCCA |
| *Gapdh* | Forward primer | CAAGGCTGAGAATGGGAAGC |
|  | Reverse primer | GAAGACGCCAGTAGACTCCA |
